# Thigh Injections of Cabotegravir + Rilpivirine in Virally Suppressed Adults With HIV-1: A Substudy of the Phase 3b ATLAS-2M Study

**DOI:** 10.1093/cid/ciae620

**Published:** 2025-01-29

**Authors:** Susan L Ford, Franco Felizarta, Kelong Han, Kehui Wang, Herta Crauwels, Anna Dari, Mar Masia, Miguel Garcia Deltoro, Olaf Degen, Jonathan B Angel, Chiu-Bin Hsiao, Carolina Acuipil, Irina Kolobova, Conn Harrington, Kelly Rimler, William Spreen, Ronald D’Amico

**Affiliations:** GSK, Durham, North Carolina, USA; Private practice of Franco Felizarta, MD, Bakersfield, California, USA; GSK, Collegeville, Pennsylvania, USA; GSK, Collegeville, Pennsylvania, USA; Janssen Research & Development, Beerse, Belgium; Janssen Research & Development, Beerse, Belgium; Infectious Diseases Section, Department of Internal Medicine, Hospital General Universitario de Elche, Elche, Alicante, Spain; Centro de Investigación en Red de Enfermedades Infecciosas, Instituto de Salud Carlos III, Madrid, Spain; Infectious Disease Service, Consorcio Hospital General Universitario de Valencia, Valencia, Spain; Outpatient Centre of the UKE GmbH, Department of Infectious Diseases, University Medical Center Hamburg–Eppendorf, Hamburg, Germany; Division of Infectious Diseases, University of Ottawa and the Ottawa Hospital Research Institute, Ottawa, ON, Canada; Positive Health Clinic, Center for Inclusion Health, Allegheny Health Network, Drexel University College of Medicine, Pittsburgh, Pennsylvania, USA; ViiV Healthcare, Durham, North Carolina, USA; ViiV Healthcare, Durham, North Carolina, USA; ViiV Healthcare, Durham, North Carolina, USA; GSK, Collegeville, Pennsylvania, USA; ViiV Healthcare, Durham, North Carolina, USA; ViiV Healthcare, Durham, North Carolina, USA

**Keywords:** cabotegravir, rilpivirine, long-acting, thigh, HIV

## Abstract

**Background:**

Cabotegravir + rilpivirine (CAB + RPV) administered via intramuscular gluteal injections is the first complete long-acting regimen for maintaining human immunodeficiency virus type 1 (HIV-1) virologic suppression. We present substudy results on short-term repeat intramuscular CAB + RPV long-acting thigh injections in participants with ≥3 years of experience with gluteal administration during the ATLAS–2M study.

**Methods:**

Substudy phases included screening, thigh injection (day 1–week 16), and return to gluteal injection (week 16–week 24). The injection schedule was unchanged from the main study. Outcomes included pharmacokinetics, safety, tolerability, efficacy, and patient-reported outcomes. Pharmacokinetic parameters were determined using noncompartmental analysis and mixed-effects modeling. Population pharmacokinetic simulations were performed.

**Results:**

There were 118 participants. In the arm that received injections every 2 months (Q2M), first CAB thigh injection including area under the concentration–time curve and maximum observed concentration (C_max_) and first RPV thigh injection C_max_ and all last RPV thigh injection parameters were statistically higher vs gluteal injections (paired comparison). No significant differences occurred with once-monthly (QM) dosing. No participants had HIV-1 RNA ≥50 copies/mL after thigh injections. Overall, 4%–7% of injection site reactions (ISRs) were grade 3. Five participants withdrew due to an ISR or injection intolerability. Overall, 30% preferred thigh vs gluteal injections. Simulations demonstrated the potential for chronic/continuous QM or ≤2 consecutive Q2M thigh injections.

**Conclusions:**

These data demonstrate the potential use of chronic/continuous QM and rotational/short-term QM or Q2M (≤4 months of continuous dosing), CAB + RPV long-acting intramuscular thigh administration for HIV-1 treatment.

## Teaser

Long-acting (LA) cabotegravir + rilpivirine (CAB + RPV) is indicated for chronic/continuous gluteal intramuscular administration. These data demonstrate the potential for chronic/continuous once-monthly (QM) and rotational/short-term QM or every-2-months CAB + RPV LA intramuscular lateral thigh administration within an established gluteal regimen.

Long-acting (LA) cabotegravir plus rilpivirine (CAB + RPV) administered once monthly (QM) or every 2 months (Q2M) via intramuscular (IM) ventrogluteal (recommended injection site) [1] or dorsogluteal injections is the first and only complete LA regimen recommended for virologically suppressed people with human immunodeficiency virus (HIV) [1–5].

CAB and RPV pharmacokinetic (PK) data following IM gluteal injections have been reported previously for both CAB LA and RPV LA separately and in combination in people with and without HIV type 1 (HIV-1) [6–10] and have been further characterized with population PK (PPK) analyses [11, 12]. Both exhibit absorption-limited (flip-flop) kinetics (the absorption rate is slower than the elimination rate). The apparent terminal half-life ranged from 3.6 to 7.7 weeks for CAB LA in studies in people without HIV [9] and 6.4 weeks from PPK models (5.6 weeks for males and 11.5 weeks for females) in both people with and without HIV [11]. For RPV LA, PPK analyses have demonstrated a terminal half-life of approximately 200 days [12]. Across phase 2/3/3b studies, CAB + RPV LA dosed every 4 weeks (Q4W) or every 8 weeks (Q8W) demonstrated median CAB LA and RPV LA IM trough plasma concentrations at the end of the dosing interval (C_τ_) that remained well above (approximately >8-fold for CAB and >3-fold for RPV) their respective protein-adjusted 90% inhibitory concentrations (PA-IC_90_: CAB, 0.166 µg/mL; RPV, 12.0 ng/mL) throughout assessment periods up to 152 weeks in people with HIV on CAB + RPV LA treatment [7, 13–16].

The thigh muscle, which has high volume capacity, could provide a potential alternative site of IM administration for participants who experience injection site fatigue or intolerability or have a contraindication for gluteal muscle administration (eg, buttock implants). The vastus lateralis (lateral thigh) muscle has been used as an administration site for other drugs and vaccines and is commonly used in children [17, 18]. Initial investigation of single-dose CAB + RPV LA IM injections to the vastus lateralis muscle in a phase 1 study of individuals without HIV supported further evaluation of thigh injections. However, PK data were suggestive of potentially faster absorption following CAB LA administration to the lateral thigh compared with the gluteus muscle, which could lead to subtherapeutic maintenance levels [19].

Here, we present the PK, safety, tolerability, antiviral activity, and patient-reported outcome measures of QM and Q2M CAB + RPV LA following short-term (across 16 weeks) repeat IM thigh administration in a substudy of adults with HIV-1 who had received ≥3 years of IM gluteal injections while participating in the ongoing phase 3b ATLAS-2M study. To further inform observed data and explore the impact of potential faster CAB LA absorption, we also present PPK simulations following chronic (CAB + RPV LA) or various dosing schedules of intermittent (CAB LA) thigh injections administered QM and Q2M to evaluate the potential for thigh administration beyond the 16-week assessment in the substudy.

## METHODS

### Study Design and Participants

ATLAS-2M (NCT03299049) is a phase 3b randomized, open-label, multicenter, parallel-group, noninferiority study that assessed the efficacy and safety of CAB + RPV LA administered Q8W vs Q4W (referred to as Q2M and QM throughout to align with the PPK modeling approach and prescribing information) in virologically suppressed adults with HIV-1. The design and eligibility criteria for the full study have been published previously [14]; the full protocol is available online [20].

The ATLAS-2M protocol was amended to allow participants to volunteer and consent for this substudy that evaluated the short-term use of CAB + RPV LA via repeat IM thigh administration. Key eligibility criteria for the substudy included participants having received CAB + RPV LA for ≥152 weeks during ATLAS-2M as well as having HIV-1 RNA <50 copies/mL at substudy screening. Key exclusion criteria included >1 virologic blip (HIV-1 RNA ≥50 to <200 copies/mL) within the 24 weeks prior to substudy screening and any suspected virologic failure (any measurement of HIV-1 RNA >200 copies/mL).

**Figure 1.**
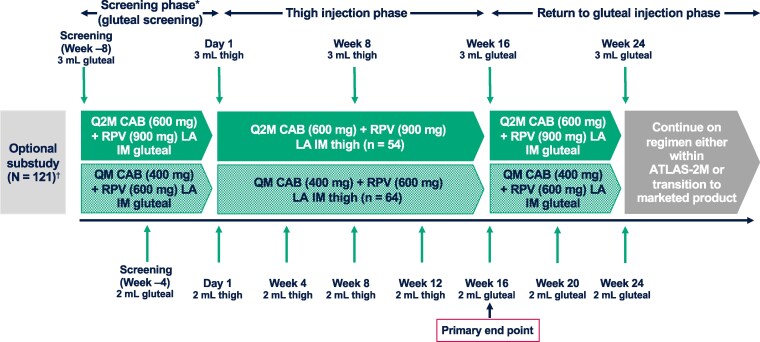
Study design. Abbreviations: ATLAS-2M, Antiretroviral Therapy as Long Acting Suppression every 2 Months; CAB, cabotegravir; IM, intramuscular; LA, long-acting; Q2M, every 2 months; QM, once monthly; RPV, rilpivirine. *Gluteal injection pre-thigh phase (control). ^†^Eligible participants had received ≥3 years of gluteal injections. There was a 20% female (sex at birth) enrollment target. Three participants failed screening.

The substudy comprised a screening phase, followed by a thigh injection phase and a return to gluteal injection phase (Figure 1). Participants continued the regimen they were randomized to on day 1 in the main study (QM or Q2M) throughout the thigh substudy. Participants received an IM injection of CAB LA (QM: 2 mL, 400 mg; Q2M: 3 mL, 600 mg) on 1 lateral thigh and RPV LA (QM: 2 mL, 600 mg; Q2M: 3 mL, 900 mg) into the opposite lateral thigh on day 1 of the thigh injection phase. Subsequent thigh injections with the same doses occurred at weeks 4, 8, and 12 for participants in the QM arm and week 8 only for the Q2M arm. Participants who completed the thigh injection phase resumed IM gluteal injections at week 16.

### Objectives and End Points

In the primary analysis, the PK parameters after thigh injections were compared with those following gluteal injections to determine the PK parameter ratio of the geometric least squares mean of thigh injection vs gluteal injection and associated 90% confidence intervals (CIs) within each arm (QM, Q2M) and for each drug (CAB, RPV).

Secondary outcomes included safety and tolerability; the proportion of participants with plasma HIV-1 RNA ≥50 copies/mL and <50 copies/mL (per the US Food and Drug Administration's Snapshot algorithm); incidence of confirmed virologic failure (CVF, 2 consecutive measurements of HIV-1 RNA ≥200 copies/mL); participant-reported preference for thigh injection or their previous gluteal injection; treatment satisfaction, assessed using the 12-item HIV Treatment Satisfaction Questionnaire status version (HIVTSQs); and tolerability of injections via participant-reported maximum level of pain following injections, assessed using the Numerical Rating Scale (NRS).

PPK modeling and simulations were performed to evaluate the potential for CAB and RPV LA thigh administration beyond the short-term (16-week) substudy.

### Procedures

PK samples for Q2M dosing were collected as follows: pre-dose at screening (week −8), day 1, and weeks 8 and 16; 2 hours post-dose on day 1 and week 8; 1 week post-dose at weeks −7, 1, and 9; and 4 weeks post-dose at weeks −4, 4, and 12. PK samples for QM dosing were collected pre-dose at screening (week −4), day 1, and weeks 4, 8, 12, and 16; 2 hours post-dose at day 1 and weeks 4, 8, and 12; and 1 week post-dose at weeks −3, 1, and 13. Procedures for bioanalysis of plasma CAB and RPV concentrations were consistent with those presented previously [19].

Safety assessments were made at each visit. Snapshot efficacy was assessed at day 1, week 4, week 8, week 12, and week 16. Injection site preference was assessed at weeks 8 and 24 in the Q2M arm and weeks 12 and 20 in the QM arm. At weeks 20 and 24, whether participants’ selected preferences from weeks 8 and 12 were maintained upon return to gluteal injections was investigated. Treatment satisfaction was assessed using the 12-item HIVTSQs (validated in the Long-Acting Antiretroviral Treatment Enabling Trial 2 [LATTE-2] study) [7] at day 1 and week 16 (7-point Likert scale ranging from 6, “very satisfied,” to 0, “very dissatisfied”). Scores from items 1–11 are combined to give a summary score (0, minimum; 66, maximum). The NRS was used to measure participant-reported maximum level of pain following injections and was administered to participants in the Q2M arm for thigh injections at day 1 and weeks 1, 8, and 9 and following gluteal injections at weeks −8, −7, 16, and 17; it was administered to participants in the QM arm for thigh injections at day 1 and weeks 1, 12, and 13 and for gluteal injections at weeks −4, −3, 16, and 17.

### Statistical Analyses

No formal statistical hypotheses were tested. The planned sample size of 41 participants per treatment arm would provide 80% power with an alpha of 0.1 and would be sufficient to evaluate the geometric mean ratio (thigh injection [test] vs gluteal injection [reference]) of PK parameters and to assess safety parameters within 16 weeks of the thigh injection phase. CAB and RPV PK parameters were determined by noncompartmental analysis (linear-up/log-down application of the trapezoidal rule), including area under the concentration–time curve from time 0 to last quantifiable time point (AUC_(0–τ)_), maximum observed concentration (C_max_), and concentration at the end of the dosing interval (C_τ_). CAB and RPV PK parameters following thigh administration were compared with those following gluteal administration using mixed-effects modeling. PK parameters were summarized using an analyte and treatment regimen using descriptive statistics. Where appropriate, an estimation approach was taken, and point estimates and 90% CIs were constructed. Comparisons were considered statistically significant if the 90% CI for geometric least squares mean ratio fell outside of the 0.8–1.25 bioequivalence criteria range; only paired data were included. Adverse events (AEs), efficacy, and patient-reported outcome measures were summarized descriptively.

### PPK Analyses and Simulations

In order to evaluate the potential for thigh administration beyond 16 weeks, established CAB (oral + gluteal IM) and RPV (gluteal IM) PPK models [11, 12] were modified as described in the Supplementary Material, page 1 [21, 22]. CAB and RPV PK profiles following chronic thigh injections administered QM and Q2M were simulated in 5000 virtual participants using respective CAB and RPV thigh PPK models (Supplementary Material, page 1). For CAB, various intermittent dosing schedules were also simulated, comprising 1-thigh–1-gluteal injection (1 thigh injection followed by 1 gluteal injection, with this pattern continuing thereafter), with equivalent schedules simulated for 2-thigh–2-gluteal injections and 3-thigh–3-gluteal injections. The PK benchmarks were selected to maintain concentrations in 95% of participants above 0.45 μg/mL for CAB and 17.3 ng/mL for RPV, the fifth percentiles of observed C_τ_ concentrations following gluteal initiation injections in phase 3 studies [16, 23].

ATLAS-2M was conducted in accordance with the Declaration of Helsinki and the Council for International Organizations of Medical Sciences’ international ethical guidelines. Written informed consent was obtained from all participants. The study protocol, amendments, informed consent, and other information were reviewed and approved by an institutional review board/independent ethics committee.

## RESULTS

### Disposition and Participant Characteristics

Overall, 121 participants from ATLAS-2M were screened for the substudy, and 118 participants were enrolled (Q2M, n = 54; QM, n = 64). Median age (range) was 48 years (24–71), 38% (n = 45 of 118) were female (sex at birth), and the median (range) body mass index (BMI) was 25 kg/m^2^ (17.9–52.7; ≥30 kg/m^2^, n = 27 of 118 [23%]). Baseline characteristics were similar between arms (Table 1).

**Table 1.** Baseline Characteristics for the Substudy at Baseline

Characteristic	Every 2 Months (n = 54)	Once Monthly (n = 64)	Total (N = 118)^a^
Median age, (range), y	50 (24–71)	46 (26–65)	48 (24–71)
Female (sex at birth), n (%)	19 (35)	26 (41)	45 (38)
Male (sex at birth), n (%)	35 (65)	38 (59)	73 (62)
Race, n (%)			
White	43 (80)	54 (84)	97 (82)
Black or African American	11 (20)	7 (11)	18 (15)
Other races	0	3 (5)	3 (3)
Hispanic/Latinx, n (%)	3 (6)	8 (13)	11 (9)
Median body mass index, kg/m^2^ (interquartile range; range)	25.5 (23.2–29.6; 17.9–51.0)	25.2 (23.0–29.9; 19.6–52.7)	25.4 (23.2–29.7; 17.9–52.7)

^a^Six participants (n = 3 in each arm) withdrew during the substudy and returned to gluteal injections. Reasons for withdrawal in the every-2-months arm were injection intolerability (n = 1), frequency of visits and injection intolerability (n = 1), and adverse event (injection site pain; n = 1). Reasons for withdrawal in the once-monthly arm were injection intolerability (n = 2) and the participant being unable to participate in 2-hour post-dose study procedures due to work schedule (n = 1).

### Pharmacokinetics

#### PK Parameters From the Substudy

**Figure 2.**
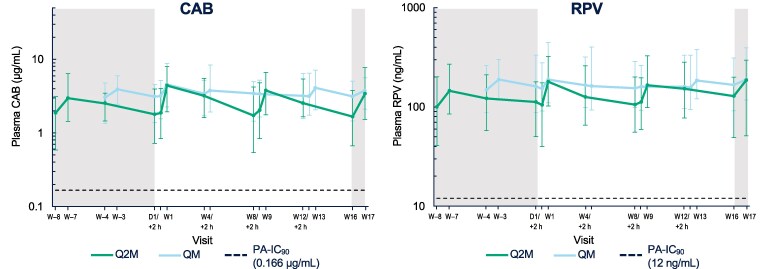
Median (5th, 95th percentiles) plasma CAB and RPV concentration–time plots. Shaded areas represent the gluteal injection phases. Abbreviations: CAB, cabotegravir; D, day; PA-IC_90_, protein-adjusted 90% inhibitory concentration; Q2M, every 2 months; QM, once monthly; RPV, rilpivirine; W, week.

**Figure 3.**
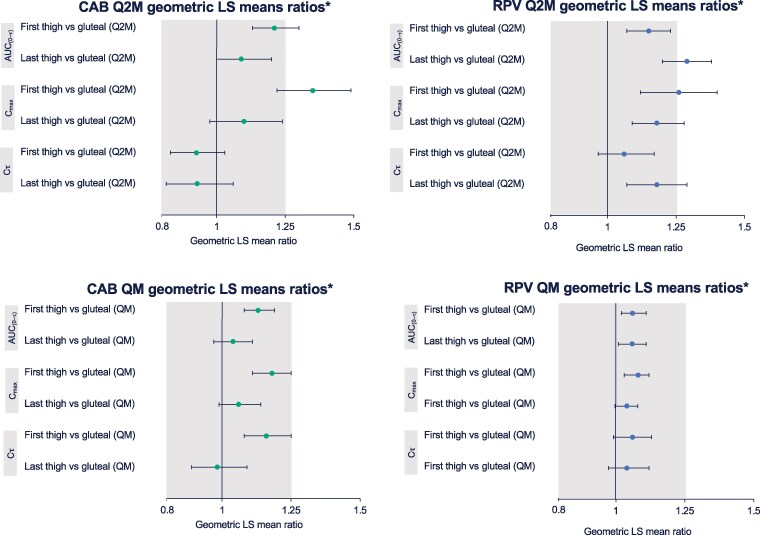
CAB and RPV geometric LS mean ratios following IM thigh and gluteal administration. Abbreviations: AUC_(0–τ)_, area under the concentration–time curve from time 0 to last quantifiable time point; C_τ_, concentration at the end of the dosing interval; CAB, cabotegravir; C_max_, maximum observed concentration; IM, intramuscular; LS, least squares; Q2M, every 2 months; QM, once monthly; RPV, rilpivirine. *Significance was determined when the 90% confidence intervals of the geometric mean ratio fell outside of the 0.8–1.25 range. The gluteal injection during the screening phase prior to switching to thigh injections was used as the reference parameter.

Plasma C_τ_ remained above the CAB and RPV PA-IC_90_ values throughout the thigh injection phase for both regimens (Figure 2). In the Q2M arm, the first CAB thigh injection AUC_(0–τ)_ and C_max_, first RPV thigh injection C_max_, and all last RPV thigh injection parameters were statistically higher vs gluteal injections (Figure 3, Table 2). No statistically significant differences in PK parameters occurred in the QM arm. Two hours following the second thigh injection, 1 participant (Q2M arm) had an observed plasma CAB concentration of 60.9 μg/mL, which met the project-defined high 2-hour PK criterion (>22.5 μg/mL) and was consistent with potential inadvertent partial intravenous administration. The corresponding RPV concentration was 80.8 ng/mL, which was similar to the pre-dose RPV concentration of 84 ng/mL (see Supplementary Material, page 2, for further details on this participant).

**Table 2.** Cabotegravir and Rilpivirine Pharmacokinetics Geometric Least Squares Means Following Thigh and Gluteal Administration

Agent	Regimen	Parameter	First Thigh	Gluteal (Paired)	First Thigh vs Gluteal^a^	Last Thigh	Gluteal (Paired)	Last Thigh vs Gluteal^a^
Cabotegravir	Q2M	AUC_(0–τ)_ (μg × h/mL)	4062	3354	1.21 [1.13, 1.30] (28.2%) [n = 41]	3416	3124	1.09 [1.00, 1.20] (26.4) [n = 32]
		C_max_ (μg/mL)	4.52	3.36	**1.35 [1.22, 1.49]** (22.8%) [n = 54]	3.70	3.36	1.10 [0.975, 1.24] (19.2%) [n = 50]
		C_τ_ (μg/mL)	1.70	1.83	0.926 [0.831, 1.03] (58.3%) [n = 30]	1.62	1.74	0.929 [0.816, 1.06] (48.1%) [n = 36]
	QM	AUC_(0–τ)_ (μg × h/mL)	2504	2210	1.13 [1.08, 1.19] (32.3%) [n = 48]	2343	2259	1.04 [0.97, 1.11] (27.5%) [n = 50]
		C_max_ (μg/mL)	4.64	3.93	1.18 [1.11, 1.25] (31.1%) [n = 60]	4.09	3.86	1.06 [0.989, 1.14] (25.5%) [n = 59]
		C_τ_ (μg/mL)	3.26	2.81	1.16 [1.08, 1.25] (30.8%) [n = 44]	2.90	2.95	0.983 [0.890, 1.09] (23.7%) [n = 43]
Rilpivirine	Q2M	AUC_(0–τ)_ (ng × h/mL)	184 311	160 755	1.15 [1.07, 1.23] (29.2%) [n = 41]	205 973	160 178	**1.29 [1.20, 1.38]** (28.0%) [n = 33]
		C_max_ (ng/mL)	184	146	**1.26 [1.12, 1.40]** (27.2%) [n = 54]	172	146	**1.18 [1.09, 1.28]** (45.3%) [n = 51]
		C_τ_ (ng/mL)	109	103	1.06 [0.966, 1.17] (37.6%) [n = 30]	123	104	**1.18 [1.07, 1.29]** (35.7%) [n = 26]
	QM	AUC_(0–τ)_ (ng × h/mL)	121 245	114 073	1.06 [1.02, 1.11] (31.5%) [n = 49]	123 246	116 112	1.06 [1.01, 1.11] (31.6%) [n = 50]
		C_max_ (ng/mL)	212	197	1.08 [1.03, 1.12] (31.4%) [n = 61]	205	197	1.04 [0.997, 1.08] (31.2%) [n = 59]
		C_τ_ (ng/mL)	167	159	1.06 [0.991, 1.13] (30.5%) [n = 44]	168	161	1.04 [0.974, 1.12] (35.0%) [n = 43]

Individuals with both test and reference (thigh and gluteal; paired data) parameters were included in geometric least squares mean (GLSM) ratio calculations. The gluteal injection during the screening phase prior to switching to thigh injections was used as the reference parameter.

Abbreviations: AUC_(0–τ)_, area under the concentration–time curve from time 0 to last quantifiable time point; C_τ_, concentration at the end of the dosing interval; C_max_, maximum observed concentration; Q2M, every 2 months; QM, once monthly.

^a^GLSM ratios (90% confidence intervals [CIs], within-subject coefficient of variation, and n values) are shown. Bolded numbers are statistically significant. Significance was determined when the 90% CIs of the GLSM ratio fell outside of the 0.8–1.25 range. n values represent paired data.

#### PPK Simulations

**Figure 4.**
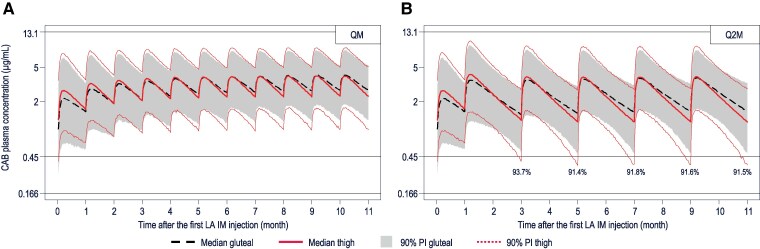
Simulated chronic CAB QM (*A*) and Q2M (*B*) administration to the lateral thigh and gluteal muscles. Simulations were performed in a population of 25% females (sex at birth). Percentages represent the proportions of simulated participants with trough plasma concentration at the end of the dosing interval (C_τ_) >0.45 µg/mL at the time points for which the percentage was simulated to be <95%. Reference lines: 0.166 µg/mL = protein-adjusted 90% inhibitory concentration; 0.45 µg/mL = 5th percentile of observed C_τ_ following the gluteal initiation injection in phase 3 treatment studies; 13.1 µg/mL = median steady-state maximum plasma concentration (C_max_) following daily administration of oral CAB 60 mg observed in study LAI116482 (Long-Acting Antiretroviral Treatment Enabling Trial [LATTE]) without dose-limiting toxicity, which was the highest C_max_ ever observed in CAB long-term studies in adults. Abbreviations: CAB, cabotegravir; gluteal, gluteal injection; IM, intramuscular; LA, long-acting; PI, prediction interval; Q2M, every 2 months; QM, once monthly; thigh, thigh injection.

**Figure 5.**
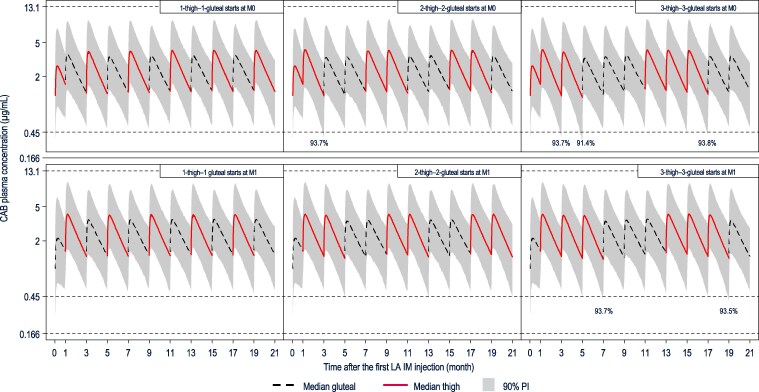
Simulated various doses of intermittent CAB Q2M administration to the lateral thigh and gluteal muscles. Simulations were performed in a population of 25% females (sex at birth). Percentages represent the proportions of simulated participants with trough plasma concentration at the end of the dosing interval (C_τ_) >0.45 µg/mL at the time points for which the percentage was simulated to be <95%. Reference lines: 0.166 µg/mL = protein-adjusted 90% inhibitory concentration; 0.45 µg/mL = 5th percentile of observed C_τ_ following the gluteal initiation injection in phase 3 treatment studies; 13.1 µg/mL = median steady-state maximum plasma concentration (C_max_) following daily administration of oral CAB 60 mg observed in study LAI116482 (Long-Acting Antiretroviral Treatment Enabling Trial [LATTE]) without dose-limiting toxicity, which was the highest C_max_ ever observed in CAB long-term studies in adults. Abbreviations: CAB, cabotegravir; gluteal, following gluteal injection; IM, intramuscular; LA, long-acting; M, month; PI, prediction interval; Q2M, every 2 months; QM, once monthly; thigh, thigh injection.

**Figure 6.**
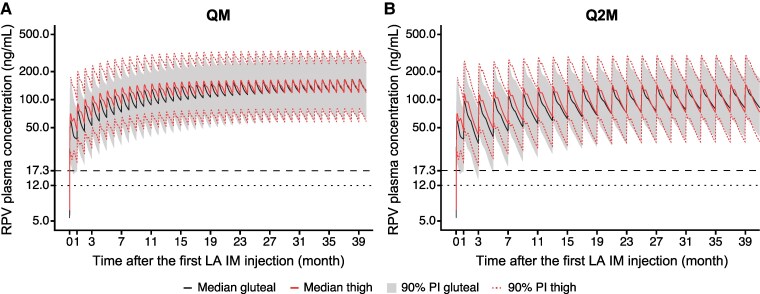
Simulated chronic RPV QM (*A*) and Q2M (*B*) administration to the lateral thigh and gluteal muscles. Reference lines: 12 ng/mL = protein-adjusted 90% inhibitory concentration; 17.3 ng/mL = 5th percentile of observed trough plasma concentration at the end of the dosing interval following the gluteal initiation injection in phase 3 treatment studies. Abbreviations: gluteal, gluteal injection; IM, intramuscular; LA, long-acting; PI, prediction interval; Q2M, every 2 months; QM, once monthly; RPV, rilpivirine; thigh, thigh injection.

Based on simulations, the CAB PK benchmark (95% of participants maintain C_τ_ >0.45 μg/mL) was achieved for chronic QM thigh injections of CAB LA but not following chronic Q2M thigh injections (<92% of participants maintained >0.45 μg/mL; Figure 4). The PK benchmark was maintained following alternating thigh and gluteal injections for both QM and Q2M regimens of up to 2 consecutive thigh injections (Figure 5). However, C_τ_ following a third consecutive Q2M thigh injection was simulated to stay above 0.45 µg/mL in <95% (91%–94%) of participants. The RPV PK benchmark (95% of participants maintain C_τ_ >17.3 ng/mL) was achieved for chronic QM and Q2M thigh injections of RPV LA (Figure 6).

### Safety

The AE profile of CAB + RPV LA thigh injections was similar between dosing arms (Table 3). Overall, AEs occurred in 80% (n = 94 of 118) of participants, and drug-related AEs occurred in 69% (n = 82 of 118) of participants; most were attributable to injection site reactions (ISRs), with 3% (n = 4 of 118) of participants experiencing a non-ISR, drug-related AE. Excluding ISRs, drug-related AEs (event level) were pyrexia (n = 2), feeling hot, nasopharyngitis, odynophagia, arthralgia, headache, choking sensation, and flushing (all n = 1); all were grade 1 in severity. No serious AEs occurred, and no participants withdrew due to a non-ISR AE.

**Table 3.** Safety Summary for the Thigh Injection Phase

Parameter, n (%)	Every 2 Months (n = 54)	Once Monthly (n = 64)	Total (N = 118)
Any AE	45 (83)	49 (77)	94 (80)
Excluding ISRs	26 (48)	26 (41)	52 (44)
Any drug-related AE	39 (72)	43 (67)	82 (69)
Excluding ISRs	2 (4)	2 (3)	4 (3)
Grade ≥3 AE	4 (7)	9 (14)	13 (11)
Excluding ISRs	0	4 (6)	4 (3)
Any drug-related grade ≥3 AE^a^	4 (7)	6 (9)	10 (8)
Serious AEs	0	0	0
AEs that led to withdrawal	1 (2)^b^	0	1 (<1)^b^

Abbreviations: AE, adverse event; ISR, injection site reaction.

^a^All were injection site pain.

^b^Grade 2 injection site pain.

ISRs occurred in 70% (n = 83 of 118) of participants. Overall, 327 (Q2M, n = 132; QM, n = 195) ISRs occurred across 704 injections (Q2M, n = 210; QM, n = 494). Injection site pain was the most common ISR, occurring with 52% (n = 110 of 210) of injections in the Q2M arm and 33% (n = 163 of 494) of injections in the QM arm. All other ISR types occurred with <3% of injections in each dosing arm (Table 4). Most ISRs were grade 1 or 2 (Q2M, 93% [n = 123 of 132]; QM, 96% [n = 187 of 195]), with few grade 3 events (Q2M, 7% [n = 9 of 132]; QM, 4% [n = 8 of 195]; no grade 4 or 5 ISRs occurred. The median (interquartile range) duration of ISRs was 3 days (2–5). One (<1%) participant withdrew due to an ISR (Q2M; grade 2 injection site pain); an additional 2 participants in each arm withdrew, citing injection intolerability as a reason. See Supplementary Material, page 2, for an AE overview of the participant with potential inadvertent partial intravenous administration.

**Table 4.** Injection Site Reaction Event-Level Safety Overview for the Thigh Injection Phase

Parameter	Every 2 Months (n = 54)	Once Monthly (n = 64)	Total (N = 118)
Number of injections	210	494	704
ISR events, n	132	195	327
Injection site pain, n (% of injections)^a^	110 (52)	163 (33)	273 (39)
Injection site discomfort, n (% of injections)^a^	6 (3)	8 (2)	14 (2)
Injection site induration, n (% of injections)^a^	4 (2)	5 (1)	9 (1)
Injection site swelling, n (% of injections)^a^	2 (<1)	5 (1)	7 (1)
Injection site erythema, n (% of injections)^a^	3 (1)	2 (<1)	5 (<1)
Grade 3 ISR events, n (% of ISRs)^b^	9 (7)	8 (4)	17 (5)
Median duration, (interquartile range), days	3.5 (3.0, 5.0)	3.0 (2.0, 4.0)	3.0 (2.0, 5.0)
Withdrawal due to injection-related reasons^c^	3 (6)	2 (3)	5 (4)

Abbreviations: ISR, injection site reaction; Q2M, every 2 months.

^a^Those that occurred with ≥1% of injections in either arm are shown.

^b^All were injection site pain events. No grade 4 or 5 ISRs occurred.

^c^Includes 1 participant who discontinued due to an ISR adverse event (every-2-months [Q2M] arm) and 4 additional participants who withdrew from the study citing injection intolerability (Q2M, n = 2; once monthly, n = 2).

### Efficacy

No participants had CVF or plasma HIV-1 RNA ≥50 copies/mL after they received thigh injections during the substudy. Similarly, high rates of virologic suppression were observed across both arms (Q2M, 94% [n = 51 of 54]; QM, 95% [n = 61 of 64]) at the substudy week 16 Snapshot. Three participants in each arm had no virologic data (discontinuation due to AE [Q2M, n = 1]; discontinuation due to other reasons [Q2M, n = 2; QM, n = 3]).

### Patient-Reported Outcomes

#### Preference

**Figure 7.**
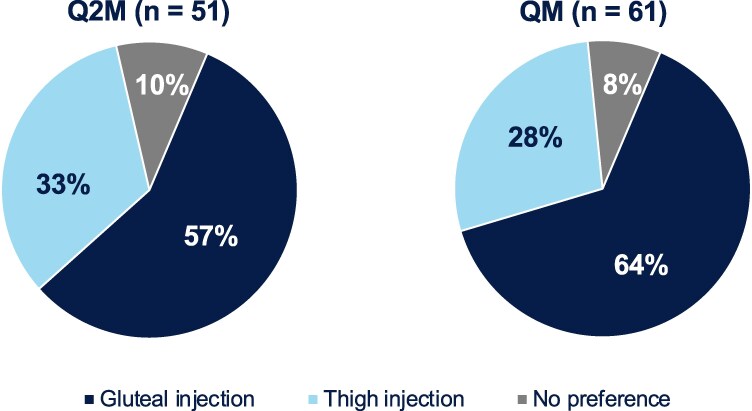
Preference for thigh injection vs gluteal injection. Assessed via a questionnaire presented at the return-to-gluteal-injection phase. Abbreviations: Q2M, every 2 months; QM, once monthly.

Overall, of participants who responded to the preference questionnaire at week 20 (QM) and week 24 (Q2M), 30% (Q2M, 33% [n = 17 of 51]; QM, 28% [n = 17 of 61]) preferred thigh injections vs 61% who preferred gluteal injections (Q2M, 57% [n = 29 of 51]; QM, 64% [n = 39 of 61]); the remaining 9% (n = 10 of 112) of participants expressed no preference (Figure 7). The most common reasons for each preference are provided in Supplementary Material, page 2.

#### HIVTSQs

HIVTSQs mean (standard deviation [SD]) total scores were high at substudy baseline and comparable between arms (Q2M [n = 54], 63.85 [3.38]; QM [n = 64], 64.03 [3.38]). Mean (SD) HIVTSQs total scores numerically decreased slightly in both dosing arms during the thigh injection phase from baseline to week 16 (Q2M [n = 51], 61.69 [6.91]; QM [n = 61], 61.74 [8.79]), returning close to baseline levels at week 24 during the return to gluteal phase (Q2M [n = 51], 63.78 [3.74]; QM [n = 61], 63.25 [4.91]).

#### Tolerability of Injections

**Figure 8.**
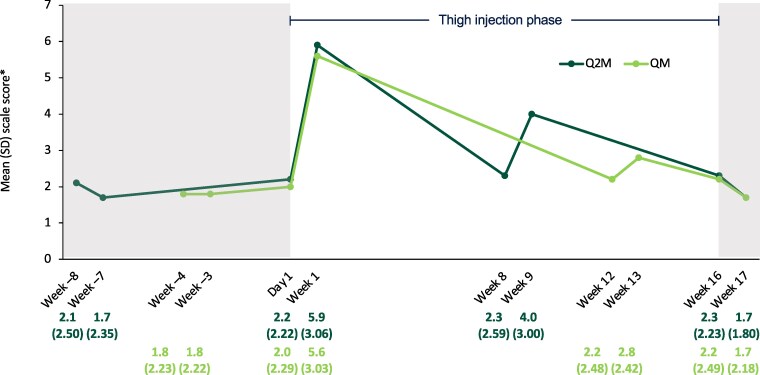
Tolerability of injections (Numerical Rating Scale). Shaded areas represent the gluteal injection phases. Abbreviations: Q2M, every 2 months; QM, once monthly; SD, standard deviation. *Scored from 0, “no pain,” to 10, “extreme pain.”

Mean pain scores, as assessed using the NRS, were numerically higher 1 week after thigh injections compared with after gluteal injections in the screening phase and in the return to gluteal injection phase. Post-thigh injection pain scores decreased over subsequent injections and were similar between arms (Figure 8).

## DISCUSSION

Together with the results from the previous phase 1 study that evaluated single-dose CAB + RPV LA IM injections to the lateral thigh in adults without HIV [19], the results of this substudy of people with HIV with ≥3 years of experience with CAB + RPV LA gluteal injections, complemented by PPK simulations, demonstrate the potential for rotational/short-term (up to 4 months of continuous dosing) CAB + RPV LA IM lateral thigh administration as an alternative site of injection within an established gluteal regimen for individuals unable to receive gluteal injections during selected treatment periods.

Statistically significant (though not clinically relevant) increases in CAB C_max_ (relative to gluteal injections), with no impact on C_τ_, following 16 weeks of thigh injections (2 Q2M doses or 4 QM doses) are indicative of an increased rate of absorption and lead to a reduced steady-state C_τ_ (C_τ–SS_). This observation is consistent with data from an earlier study, which suggested faster absorption with thigh CAB injections vs gluteal injections [19]. Furthermore, in participants who received CAB + RPV LA Q2M without prior CAB + RPV exposure in ATLAS-2M, RPV C_τ_ at week 152 was twice as high as C_τ_ at week 8, while CAB C_τ_ at week 152 was similar to C_τ_ at week 8 [15]. Owing to a lack of CAB accumulation, reduced CAB C_τ-SS_ following Q2M CAB thigh injections may fall below the C_τ-SS_ benchmark observed in phase 3 studies of gluteal injections, potentially resulting in reduced efficacy. CAB PPK modeling was consistent with the increased absorption rate for thigh injections. PPK analyses have demonstrated slower absorption of CAB LA in females and participants with high BMI for both gluteal and thigh administration [11, 21]. By a similar mechanism, the faster absorption of CAB LA with thigh administration is potentially due to the thinner subcutaneous fat layer and higher vascularity in the thigh vs buttocks [21].

PPK simulations demonstrated that the CAB PK benchmark was achieved for chronic QM thigh injections but not for chronic Q2M thigh injections of CAB LA. For RPV, chronic QM and Q2M thigh injections both met the PK benchmark. Therefore, for the treatment of HIV-1, the potential for chronic thigh administration is limited to QM dosing of CAB + RPV LA due to the increased rate of absorption of CAB LA relative to gluteal administration.

CAB + RPV LA injections to the lateral thigh were well tolerated in the ATLAS-2M substudy, with an AE profile consistent with the phase 3/3b gluteal injection studies and no new safety signals. Notably, no serious AEs were reported, with only 1 AE leading to withdrawal (injection site pain). Consistent with the known phase 3/3b study data for gluteal administration, ISRs were the most frequent AE of thigh administration, with injection site pain being the most frequently occurring AE [13, 14, 16]. Most ISRs were grade 1 or 2 and short in duration, consistent with data from a pooled analysis of ISRs across the phase 3/3b program of gluteal administration [24].

Rates of virologic suppression after the thigh injection phase at the week 16 Snapshot were high and similar to those observed in the overall ATLAS-2M study at week 152 [15], as well as the rest of the phase 3/3b program [13, 16, 23, 25]. During this 24-week substudy, no participants had CVF or HIV-1 RNA ≥50 copies/mL after receipt of thigh injections, demonstrating the continued maintenance of virologic suppression.

Across the phase 3/3b program to date, CAB + RPV LA QM and Q2M demonstrated greater improvements in treatment satisfaction and were preferred vs daily oral therapy regimens, including in the overall ATLAS-2M study [26, 27], highlighting the importance of developing new treatment modalities that provide benefits beyond clinical efficacy and tolerability. In this study, HIVTSQs scores were high for participants who entered the substudy and received gluteal CAB + RPV LA injections. The slight numerical decreases in HIVTSQs scores during the thigh injection phase followed by increases (returning close to substudy baseline scores) upon return to gluteal injections are mirrored by the numerical increases observed in the NRS post-injection pain scores following thigh injections compared with gluteal injections. Notably, scores decreased for the subsequent thigh injections, consistent with the observation that ISRs occurred most frequently after initial gluteal injections in phase 3/3b trials, with the incidence decreasing over time [14, 16, 23]. Two-thirds of participants preferred gluteal injections, while one-third preferred thigh injections across both dosing regimens, citing convenience/access, being less bothered by pain following injection, and being less bothered by pain during injection as the most common reasons for thigh injection preference. This suggests that thigh injections may be a preferred alternative for some individuals who receive CAB + RPV LA gluteal injections. One limitation of the substudy was the relatively small sample size, which may limit the generalizability of findings to certain subgroups. Additionally, thigh injection simulations extended beyond the short duration of the substudy (and do not replace evaluation of chronic administration in a clinical trial).

## CONCLUSIONS

Both the observed efficacy and safety data generated in this study, together with PK modeling and simulations, demonstrate the potential use of chronic/continuous QM CAB + RPV LA IM thigh administration, as well as rotational/short-term QM and Q2M dosing (up to 4 months of continuous dosing) within an established gluteal regimen, for the treatment of HIV-1. Longer-term efficacy and safety data are needed to better characterize the role of CAB + RPV LA thigh administration in the treatment of HIV-1. Thigh administration of CAB + RPV LA has not been approved by regulatory agencies.

## Supplementary Material

ciae620_Supplementary_Data

## Supplementary Data

Supplementary materials are available at *Clinical Infectious Diseases* online. Consisting of data provided by the authors to benefit the reader, the posted materials are not copyedited and are the sole responsibility of the authors, so questions or comments should be addressed to the corresponding author.

## Notes

***Author Contributions.*** F. F., M. M., M. G. D., O. D., J. B. A., and C.-B. H. participated in the conduct of the study, including the recruitment and follow-up of participants. S. L. F., K. W., H. C., A. D., C. A., I. K., K. H., C. H., K. R., W. S., and R. D.’A. participated in the analysis of the study data and the conceptualization and design of the studies. All authors were involved in the drafting and review of the manuscript and approved the final version.

***Acknowledgments.*** The authors thank everyone who contributed to the study, all study participants and their families, and the clinical investigators and their staff. We also acknowledge the contributions of Vasiliki Chounta.

***Disclaimer.*** The funders participated in the collection, analysis, and interpretation of data; in the writing of the report and in the decision to submit the manuscript for publication. All authors vouch for the accuracy and completeness of the data, data analyses, and interpretation and fidelity to the protocol, and all have approved the final manuscript for submission.

***Data sharing.*** Data-sharing requests will be considered by the management group upon written request to the corresponding author. Deidentified participant data or other prespecified data will be available subject to a written proposal and a signed data-sharing agreement.

***Financial support***. This study was funded by ViiV Healthcare and J&J Innovative Medicine. Editorial assistance was provided by Daniel Williams at Nucleus Global, funded by ViiV Healthcare.

***Potential conflicts of interest***. S. L. F., K. H., K. W., and K. R. are employees and stockholders of GSK. F. F. has received research grants and honoraria for lectures from ViiV Healthcare, AbbVie, and Gilead Sciences. H. C. and A. D. are employees of Janssen and stockholders of Johnson & Johnson. M. M. has received honoraria from and has participated in educational activities and on advisory boards for ViiV Healthcare, Janssen, and MSD and reports support for travel/meeting attendance from Janssen and MSD. M. G. D. has received grants and honoraria from and has participated on a data and safety monitoring board or advisory board for ViiV Healthcare, Janssen, Gilead Sciences, MSD, and AbbVie and reports support for travel/meeting attendance from Janssen and Gilead Sciences. J. B. A. received research contracts and honoraria for educational activities from and has participated on advisory boards for ViiV Healthcare and Gilead Sciences and has received research grant support from the Canadian Institutes for HIV Research. C.-B. H. reports grants received to their institution, consulting fees, and honoraria from ViiV Healthcare and Gilead Sciences. C. A., I. K., C. H., W. S., and R. D.’A. are employees of ViiV Healthcare and stockholders of GSK. The remaining author reports no potential conflicts.
