# Supplementary material for: Thigh Injections of Cabotegravir + Rilpivirine in Virally Suppressed Adults With HIV-1: A Substudy of the Phase 3b ATLAS-2M Study
Source: Clin Infect Dis. 2025 Jan 29;81(1):101–12. doi: 10.1093/cid/ciae620 (PMC12314501; doi:10.1093/cid/ciae620)
Supplement: ciae620_Supplementary_Data [file ciae620_supplementary_data.docx]

# Supplement

## Population PK (PPK) cabotegravir (CAB) long-acting (LA) simulations methods

The established CAB (oral + gluteal intramuscular [IM]) PPK model [1] was modified by adding the thigh injection depot compartment and was fitted to pharmacokinetic (PK) data following both thigh and gluteal IM administration from this clinical study and an earlier Phase 1 study.[2] Covariates were resampled 5000 times from the model-building data set, generating 5000 virtual participants with 25% females, which represented the treatment population observed in Phase 3 treatment studies.[3, 4] Individual PK parameters of the 5000 virtual participants were calculated using participant-specific covariates and using the population parameter estimates, participant‑specific non-linear mixed-effects modeling inter-individual errors (ETA) sampled from the distributions that are determined by the estimated variance-covariate matrix of between‑participant variability from the final model. Concentration-versus-time profiles were calculated using the individual PK parameters of the 5000 virtual participants. Residual variability (EPS) was included in the simulation. Median and 90% prediction intervals (5th and 95th percentiles) of the simulated concentration-versus-time profiles were calculated.

For simulation purposes, the first thigh injection was administered either as the first injection on Day 1 (Month 0) or at Month 1 (1 month after the first gluteal injection, i.e. the 2nd injection). Interpretations made based on the Month 1 simulations were assumed to be applicable to scenarios in which the first thigh injection was administered after Month 1.

## PPK rilpivirine (RPV) LA simulations methods

The established RPV (gluteal IM) PPK model [5] was modified by adding two parallel absorption pathways (fast and slow) for thigh injections, based on data from this clinical study and an earlier Phase 1 study.[2] There are no specific covariates included in the RPV LA PPK model.[2, 5] Individual PK parameters of 5000 randomly sampled virtual participants were calculated. Concentration-versus-time profiles were calculated using the individual PK parameters of the 5000 virtual participants. Median and 90% prediction intervals (5th and 95th percentiles) of the simulated concentration-versus-time profiles were calculated. Residual variability (EPS) was included in the simulation.

Simulations were performed for either all injections as of the first injection administered in the thigh or all injections as of the first injection administered in the gluteal muscle. Simulations were performed without an oral lead-in.

## Adverse event (AE) overview of the participant with potential inadvertent partial intravenous administration

The participants (every 2 months arm) experienced three Grade 1 AEs (choking sensation, feeling hot, and flushing) on the same day as the high CAB concentration, which were considered to be related to the study intervention. The reaction was reported as occurring within seconds of the CAB injection; the RPV injection was administered 7 minutes after the CAB injection, after symptoms had resolved. While a possible post-injection reaction following CAB administration cannot be ruled out, the investigator considered the AEs to be a vasovagal reaction related to the use of a needle, with an operation for myopia 3 days prior as a contributing factor. No relevant changes in blood pressure, heart rate, or oxygen saturation were observed. Additionally, the participant had also experienced Grade 1 AEs with gluteal injections on three separate occasions in the main study, specifically reporting hot flush 3 days after the Week 64 visit, erythema and feeling warm on the day of the Week 104 visit, and feeling hot on the day of the Week 176 visit. The participant did not experience confirmed virologic failure or any subsequent viral blips during the substudy.

## Reasons for thigh injection vs. gluteal injection preference

The most common reasons for preferring thigh injections were convenience/easy access (both arms, 71% [n=12/17]), being less bothered by pain following injection (every 2 months [Q2M], 65% [n=11/17]; every 1 month [QM], 59% [n=10/17]), and being less bothered by pain during injection (Q2M, 47% [n=8/17]; QM, 65% [n=11/17]). The most common reasons for preferring gluteal injections were being less bothered by pain following injection (Q2M, 66% [n=19/29]; QM, 64% [n=25/39]), being less bothered by pain during injection (Q2M, 72% [n=21/29]; QM, 46% [n=18/39]), and being less bothered by muscular pain/stiffness when walking or doing physical activity (Q2M, 41% [n=12/29]; QM, 49% [n=19/39]).

1. Han K, Baker M, Lovern M, et al. Population pharmacokinetics of cabotegravir following administration of oral tablet and long-acting intramuscular injection in adult HIV-1-infected and uninfected subjects. British journal of clinical pharmacology **2022**; 88(10): 4607-22.

2. Han K, Gevorkyan H, Sadik Shaik J, et al. Pharmacokinetics and tolerability of cabotegravir and rilpivirine long-acting intramuscular injections to the vastus lateralis (lateral thigh) muscles of healthy adult participants. Antimicrobial Agents and Chemotherapy **2024**; 68(1): e0078123.

3. Orkin C, Arasteh K, Górgolas Hernández-Mora M, et al. Long-Acting Cabotegravir and Rilpivirine after Oral Induction for HIV-1 Infection. The New England journal of medicine **2020**; 382(12): 1124-35.

4. Swindells S, Andrade-Villanueva JF, Richmond GJ, et al. Long-Acting Cabotegravir and Rilpivirine for Maintenance of HIV-1 Suppression. The New England journal of medicine **2020**; 382(12): 1112-23.

5. Neyens M, Crauwels HM, Perez-Ruixo JJ, Rossenu S. Population pharmacokinetics of the rilpivirine long-acting formulation after intramuscular dosing in healthy subjects and people living with HIV. J Antimicrob Chemother **2021**; 76(12): 3255-62.
